# Addressing challenges with systematic review teams through effective communication: a case report

**DOI:** 10.5195/jmla.2021.1222

**Published:** 2021-10-01

**Authors:** Linda C. O'Dwyer, Q. Eileen Wafford

**Affiliations:** 1 l-odwyer@northwestern.edu, Head of Research and Information Services, Galter Health Sciences Library and Learning Center, Feinberg School of Medicine Northwestern University, Chicago, IL; 2 q-wafford@northwestern.edu, Research Librarian, Galter Health Sciences Library and Learning Center, Feinberg School of Medicine Northwestern, Chicago, IL

**Keywords:** systematic review, communication, expectations, team management, process management, project management

## Abstract

**Background:**

Every step in the systematic review process has challenges, ranging from resistance by review teams to adherence to standard methodology to low-energy commitment to full participation. These challenges can derail the project and result in significant delays, duplication of work, and failure to complete the review. Communication during the systematic review process is key to ensuring it runs smoothly and is identified as a core competency for librarians involved in systematic reviews.

**Case Presentation:**

This case report presents effective communication approaches that our librarians employ to address challenges encountered while working with systematic review teams. The communication strategies we describe engage teams through information, questions, and action items and lead to productive collaborations with publishable systematic reviews.

**Conclusions:**

Effective communication with review teams keeps systematic review projects moving forward. The techniques covered in this case study strive to minimize misunderstandings, educate collaborators, and, in our experience, have led to multiple successful collaborations and publications. Librarians working in the systematic review space will recognize these challenges and can adapt these techniques to their own environments.

## BACKGROUND

Galter Health Sciences Library and Learning Center has supported systematic reviews since 2014 with both consultation and collaboration support models. The consultation model is a one-hour meeting where the librarian provides an overview of the process and advice on the search strategy. The review team performs their own searches and other related tasks with minimal input and no expectation of coauthorship for the librarian. Under the collaboration model, the librarian assists with the protocol, search strategy development, running the searches, removing duplicate records, and creating drafts of the PRISMA diagram as well as search methods for the manuscript. Our team consists of six librarians who have coauthored over sixty published systematic and scoping reviews and have consulted on many more. The majority of our user base, made up of faculty, residents, fellows, and students, choose our full collaboration service. At any one time, Galter librarians are each working, on average, between three and seven reviews at various stages of the process. Over the last few years, we have endeavored to streamline our process, refine best practices, and improve how we collaborate and communicate with our review teams to manage the increasing popularity of our systematic review service and see more reviews completed to submission.

Every step in the systematic review process has challenges. Similar to the experience of librarians surveyed by Nicholson et al., librarians collaborating on systematic reviews at Galter regularly encounter challenges with a review team's methodology [1]. These challenges include inappropriate or unfocused research questions, resistance to multiple reviewers, grey literature exclusion, and limited information sources. Many reviewers underestimate the time commitment for completing a systematic review, resist creating a protocol, or do not actively participate with search strategy development. These challenges can result in delays, duplication of work, and failure to complete a review. Communication during the systematic review process is key to ensuring it runs smoothly. In fact, Townsend et al. identified communication as a core competency for librarians involved in systematic reviews [2]. This case report presents effective communication approaches employed by Galter librarians to address challenges encountered while working with systematic review teams. Our approach to effective communication with teams is multifaceted. We employ templates, checklists, and documents from our Systematic Review Toolkit [3, 4]. We share knowledge gained from published sources along with personal and shared experiences to teach reviewers about systematic reviews, encourage active involvement, and form productive collaborations with publishable reviews. These approaches are also applicable to other comprehensive projects such as scoping reviews.

## CASE PRESENTATION

### Challenge: Inappropriate or unfocused review question

A common problem we encounter at the outset of a systematic review is an overly broad review question or no explicit review question but merely a topic that could produce several more specific questions. Some questions are too narrow or easily answerable with a statistics search (e.g., incidence, prevalence). From experience with supporting many review teams, we can usually spot a tricky review question immediately, though it might require some preliminary searching to identify issues. We have several discussion points to help reviewers understand the importance of a focused review question and impact on their timeline:

- Broad questions may not appeal to their targeted journal.
- Broad questions produce a massive number of results that require screening.
- Many studies may meet the inclusion criteria and require full-text screening and risk of bias assessments.

Raising these potential pitfalls often helps teams realize the impracticality of broad review questions.

We sometimes encounter questions already addressed by existing systematic reviews. Galter librarians avoid collaborating on duplicative reviews as they (a) do not contribute to scholarship and (b) will likely meet resistance during the journal submission and publication process. Teams should present reasons for duplicating an existing review, such as poor methodology of the original review or the need for an update. Alternatively, they should assure us that their review is indeed different.

We typically discuss the question in more depth using information from the library's review intake form, part of the Systematic Review Toolkit [3]. We review their population-intervention-comparison-outcome (PICO) elements and explain how broad concepts affect the results, usually with the demonstration of a preliminary search strategy. Sometimes, we ask reviewers to outline the structure of their paper with the protocol or a draft of the paper outline with the following questions to help them think about key aspects of their manuscript:

- How will they analyze and organize the results?
- What kind of data will they extract from each article?
- Will they be undertaking a meta-analysis?
- Are there certain facets they want to address that would aid in structuring the narrative?

Thinking about this information at the start helps some reviewers focus and refine their questions. For teams committed to a broad question, we suggest that the question may be more usefully answered with a scoping review and ask them if they are willing to pursue that instead. Many reviewers are indeed willing once they learn how its methodology better suits their question.

### Challenge: Resistance to using more than one reviewer

While each project should have, at minimum, two independent reviewers assessing all articles throughout the process, researchers in a hurry or with limited support may wish to forego this step.

Resistance to using more than one reviewer offers the opportunity to emphasize the importance of multiple reviewers independently screening titles, abstracts, and full text; extracting data; and assessing quality to minimize bias [5]. Systematic reviews are top-level evidence precisely *because* review teams apply rigorous methods aimed at minimizing bias. We also inform reviewers that the use of a single author will likely set off alarm bells with peer reviewers during journal submission. Knowing that taking this shortcut may place the acceptance of their manuscript in jeopardy reinforces the importance of using more than one reviewer.

### Challenge: Underestimating the time commitment

Many teams underestimate the time required to complete a systematic review. Reviewers should understand systematic reviews are long-term projects that may take between six and eighteen months to complete [6–8]. Resources such as the systematic review process diagram illustrated by Tsafnat et al., found in our Systematic Review Toolkit, help us communicate estimated time requirements for each step of a review [3, 9]. While we do not systematically track librarians' time spent on systematic reviews (though we plan to do so in future), we plan for two to four weeks to develop the search strategy, which relies on teams' responsiveness to emails and questions; seven to ten days to search the various databases; and three to four days to remove duplicate results. We present estimated screening times of twenty to thirty minutes for every 200 records to thirty to sixty seconds per citation so teams can project the hours necessary to complete the title and abstract screening. We talk about the need to allot time to full text retrieval, full text screening, data extraction, data synthesis, including quality assessment, and the final write up. We also review the time commitment to update searches before the submission and the need to reemploy the screening and analysis steps for any new results as peer reviewers may return or reject manuscripts due to outdated searches. Additionally, peer reviewers might identify a relevant article published between the time we performed the original search and submitted the manuscript. Upon clarifying the steps and time commitments, we ask if the projected timeline works for the group. Teams with tighter deadlines may opt for the consultant model or no library support.

### Challenge: Resistance to inclusion of grey literature

We have worked with many teams hesitant to incorporate grey literature in their review. Many reviewers are unfamiliar with the term *grey literature*, so we explain grey literature sources and reference our Systematic Reviews guide, which is part of the toolkit [10]. This guide lists various sources of grey literature including bibliographic databases like Embase and Scopus and online platforms such as the New York Academy of Medicine, OpenGrey, MedNar, and ClinicalTrials.gov. We take responsibility for learning about different sources of grey literature and making recommendations that are appropriate for each research question.

We maintain open and honest communication by explaining the pros and cons of grey literature. The inclusion of grey literature will increase the number of records to review but may yield few relevant studies for analysis. It also requires more time-intensive methods to find and screen it; however, its inclusion produces a fuller picture by minimizing publication bias [11, 12]. To get buy-in from reviewers, we involve them from the start by asking for key societies, websites, and resources in their field on our intake form. In our experience, manuscripts have a better chance of journal acceptance if authors exhaust every resource. For reviewers unwilling to include grey literature, we suggest noting the exclusion of grey literature as a potential limitation in the manuscript.

### Challenge: Inappropriate database selections and preferences

When it comes to the number of databases to search, reviewers usually either 1) want us to restrict the search to a limited number of databases or 2) suggest every database with any possible connection to their topic, usually drawn from another systematic review. We explain how a more targeted approach will likely yield more conclusive results than an everything-but-the-kitchen-sink strategy. Posey et al. found searching three of the major bibliographic databases would capture 97–100% of the eligible studies in their analysis of ninety-seven systematic reviews that collectively searched fifty-seven sources [13]. Furthermore, the Cochrane Collaboration recommends at a minimum to search CENTRAL, MEDLINE, and Embase for clinical trials [12]. For teams who would like to limit to MEDLINE, we go over the benefits of additional sources, especially grey literature to reduce bias.

We want teams to understand how these database selections can produce a comprehensive review and reduce the likelihood of a request for revisions or rejection from a journal's editors for database selection. After our discussion and an outline of our recommendations, taking into account redundancy, time, and efficiency, reviewers are usually willing to defer to our experience with database selection. We then refine and confirm the final list of databases with the team.

### Challenge: Resistance to protocol creation and development

We periodically encounter resistance to development of a protocol. In our conversations with review teams, we convey the importance of a protocol as a tool to plan the review and address potential challenges early in the collaboration. The PRISMA checklist also requires protocol registration information [14]. The protocol should be in place prior to the start of the project as this aligns with the goals of the systematic review process to minimize bias. However, reviewers have time to work on their protocol because PROSPERO, the systematic reviews protocols registry, permits protocol registration up to the data extraction stage. This allows some latitude to change the protocol after, for example, the pilot screen. By the time a pilot screen is complete, the final inclusion and exclusion criteria should be set. Journal editors reviewing the submitted review usually require evidence of a prepublished protocol, so authors should be able to point to the location of a published protocol [12, 14].

At Galter Library, we operate a **“**no protocol, no librarian” policy. We require teams working with Galter librarians under the collaboration model to develop and register a protocol before we proceed with the search strategy development. We discuss this requirement in the initial meeting and outline it in the Memorandum of Understanding, a document that collaborating review teams sign at the start of the project. Our Protocol Development page on the online guide includes tools to develop the protocol as well as guidance on where to deposit (e.g., PROSPERO, Open Science Framework, open access journals, or Northwestern's institutional repository). We also offer to review a team's protocol before submission and offer suggestions for revision. Explaining how the absence of a protocol may be problematic during journal peer review, the benefits of a protocol, and that our involvement is contingent on the completion of a protocol usually compels the most reluctant teams to create one.

### Challenge: Difficulties with search strategy development

Development of a comprehensive search strategy can be problematic when reviewers do not understand the process, fail to provide constructive feedback, or respond to requests for input on the search terms. Search strategy development is the librarian's domain and where our expertise is most valuable. It is an iterative process reliant on ongoing communication between the team and librarian [15]. To promote and improve this interaction, we help teams understand the components of a comprehensive search, including the role of keywords, subject headings, and Boolean and proximity operators. We work with them to generate a list of candidate search terms, which involves ensuring each term in the search strategy is as targeted as possible while still not missing any potentially relevant terminology.

We ask questions such as:

- Are there additional terms for each PICO component?
- Can we eliminate a term without harming the integrity of the review?
- Do they see potentially useful articles in the preliminary PubMed search after the addition of a suggested term?
- Which terms are bringing in the majority of the literature and are they truly adding to the search?
- Do we need to explode a MeSH term and each term below the subject heading?

Upon checking for search terms that may bring in substantive numbers of irrelevant results without contributing to the search's quality, we run searches with and without those terms and ask our teams to check a set of results with the problematic terms alone.

The inclusion of word variants, synonyms, alterative drug names, and other relevant terms often results in larger search numbers than a reviewer is expecting. Teams may insist on removing certain terms, sometimes against our recommendation. At this point, we may decide the removal of the terms damage the integrity of the review as well as the search strategy and offer to provide consult-level support so reviewers can take the search in a direction in which they are comfortable.

### Challenge: Resistance to librarian coauthorship

Some teams resist adding the librarian as a coauthor; however, our support will invariably meet the International Committee of Medical Journal Editors' standards for coauthorship [16]. We use the initial consultation to discuss Galter's two-tier support model of one-off consultation versus full collaboration so reviewers understand the depth of our contribution. For teams that elect or revert to the consultation model, we communicate the limits of our contribution as consultants. Some librarians at Galter review each step of the systematic review process using Tsafnat et al.'s diagram from the Systematic Review Toolkit [9]. Outlining the services now on the reviewers' to-do list instead of the librarian's illustrates how much we help when teams use the full-service, collaborative model.

We explicitly ask about authorship attribution at the start of the project on the intake form and on the Memorandum of Understanding found in the Systematic Review Toolkit [3]. In our experience, it is rare for potential reviewers to refuse to list us as a coauthor. However, Galter's policy is to offer consultation services rather than the full collaboration for teams that decline to acknowledge the librarian as coauthors.

### Challenge: Unresolved or ongoing issues

When our communication proves ineffective and a challenge stymies progress or compromises quality, we exercise the right to remove ourselves from the review. Exiting a review gracefully can be tricky and uncomfortable. A strategy that we have employed effectively is as follows: 1) thank the team for their input, 2) describe the process/policy where there is an issue and include a brief rationale or supporting documents, and 3) provide alternative options. Using this approach, we may communicate the following to a team that does not submit a completed protocol:

Thank you for your input. As per Galter's policy, I am only able to move forward with the review if the protocol is complete and adheres to the PRISMA-P statement. Attached are documents to help the team develop the protocol. I understand that Galter Library's parameters for supporting teams under the full-collaboration/librarian as coauthor model may not work for every team's timeline or workflow. Our consultation model is more flexible and accommodates different workflows.

Fortunately, we rarely have to exit a review, and it only happens after consultation with a librarian supervisor.

## DISCUSSION

Effective communication with review teams, identified as both a constant challenge and a required competency for librarians in previous studies, keeps systematic review projects moving forward [1, 2]. This case report explores effective communication approaches used by librarians at Galter Library to meet challenges from reviewers with limited knowledge of the systematic review process, unrealistic timelines, or who may resist the inclusion of a second reviewer or grey literature. Some teams want to take short cuts with search strategy development or database selection. Alternatively, reviewers may suggest an exhaustive list of terms and databases, which will create more work for everyone involved without producing a better quality review.

We use our knowledge and experience to facilitate discussion and understanding of the process. We present facts, examples, action items, and questions to engage the reviewers. We supplement our conversations with tools from the Systematic Review Toolkit, such as the intake form with a team's PICO details, Tsafnat et al.'s flow diagram, and the Memorandum of Understanding with information about each party's responsibilities. The Systematic Reviews Guide with descriptions of the process and support models is a great reference tool, especially when we need to address authorship [10].

Our approach to communicating with review teams is a work in progress. Our monthly Systematic Review Working Group meetings help us improve our communication and overcome obstacles through sharing experiences, receiving feedback, and learning about different communication styles. Every library, institution, and user base is different, so our approach may not work in every environment. The techniques covered in this case study help minimize misunderstandings, educate reviewers, and usually lead to successful collaborations for librarians at Galter Health Sciences Library and Learning Center. Any librarian who collaborates on systematic reviews can adapt and employ these techniques as they meet the many challenges of working with review teams.

## Data Availability

There are no data associated with this article.
